# New Roles for MicroRNAs in Old Worms

**DOI:** 10.3389/fragi.2022.871226

**Published:** 2022-04-12

**Authors:** Corrina R. Elder, Amy E. Pasquinelli

**Affiliations:** Division of Biology, University of California, San Diego, La Jolla, CA, United States

**Keywords:** microRNA, *C. elegans*, aging, insulin signaling, dietary restriction, autophagy, proteostasis.

## Abstract

The use of *Caenorhabditis elegans* as a model organism in aging research has been integral to our understanding of genes and pathways involved in this process. Several well-conserved signaling pathways that respond to insulin signaling, diet, and assaults to proteostasis have defined roles in controlling lifespan. New evidence shows that microRNAs (miRNAs) play prominent roles in regulating these pathways. In some cases, key aging-related genes have been established as direct targets of specific miRNAs. However, the precise functions of other miRNAs and their protein cofactors in promoting or antagonizing longevity still need to be determined. Here, we highlight recently uncovered roles of miRNAs in common aging pathways, as well as new techniques for the ongoing discovery of miRNA functions in aging *C. elegans*.

## Introduction

Since the discovery of long-lived *Caenorhabditis elegans* genetic mutants over 30 years ago, these nematodes have become an important tool in understanding the aging process (Mack et al., 2018). Their relatively short lifespan (∼18–20 days), genetic manipulability, and consistent display of the hallmarks of aging make them an advantageous model organism for uncovering the molecular contributions to aging (Mack et al., 2018; Son et al., 2019; Zhang et al., 2020). Through the work of numerous groups, a few key modes of regulating longevity have been found in *C. elegans,* including changes in insulin/insulin-like growth factor-1 (IGF-1) signaling (IIS), target of rapamycin (TOR) signaling, AMP-activated protein kinase (AMPK) signaling, autophagy, and diet (Kenyon, 2010; Uno and Nishida, 2016; Zhang et al., 2020). The broad conservation of the genes in these pathways suggests that new insights may be pertinent to mammalian aging (Bitto et al., 2015; Khan et al., 2019). Though, unsurprisingly, recent findings highlight the complexity of translating results from aging model organisms to human gerontology (Ukraintseva et al., 2021).

**FIGURE 1:**
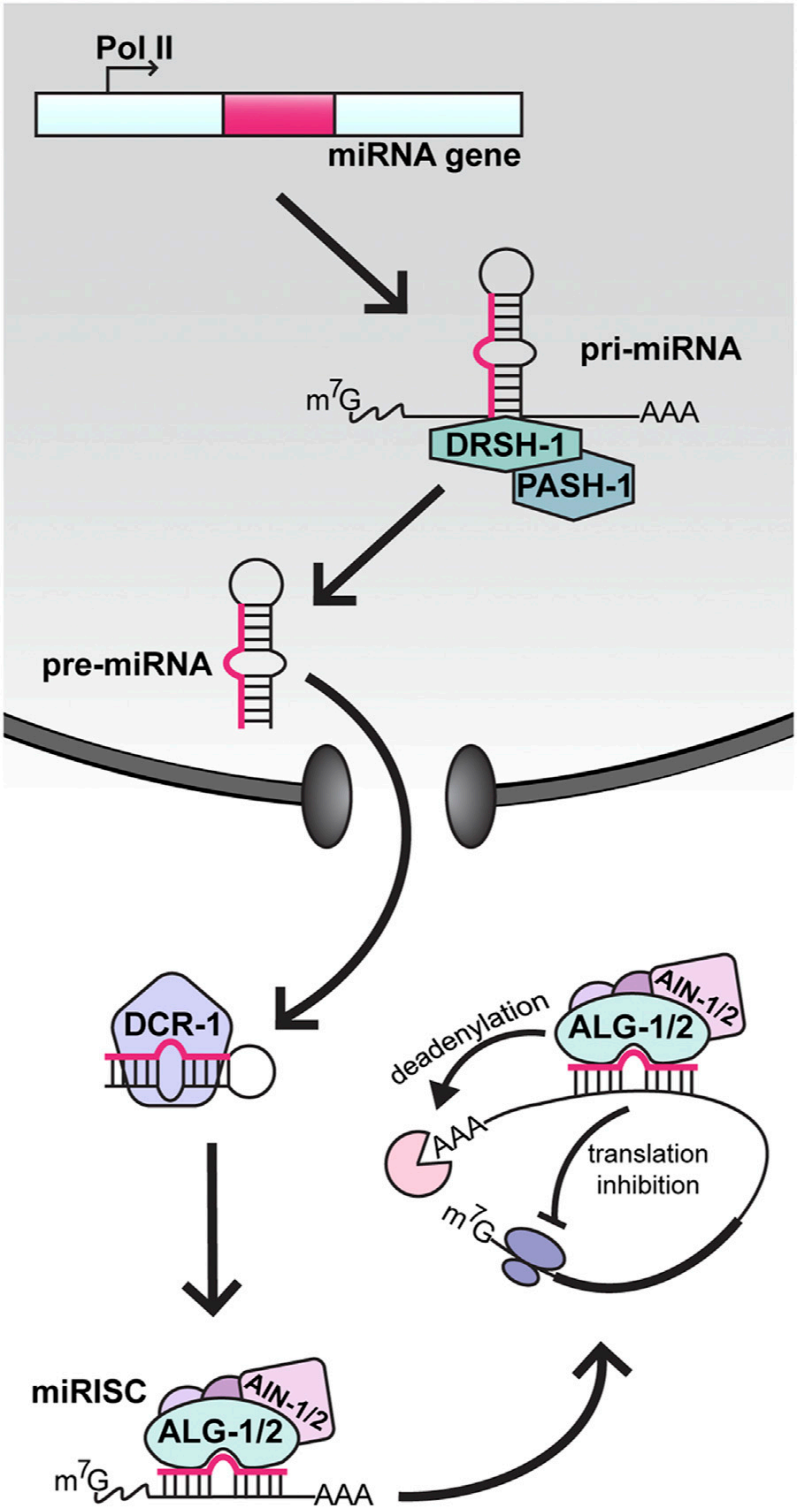
MicroRNA biogenesis and function in *C. elegans*. In the nucleus, miRNA genes are transcribed by RNA polymerase II (Pol II) into long primary transcripts (pri-miRNAs). Pri-miRNAs are cleaved by Drosha (DRSH-1) in complex with the RNA binding protein Pasha (PASH-1). The resulting precursor miRNA (pre-miRNA) hairpin is then transported to the cytoplasm, where processing by Dicer (DCR-1) produces a double stranded heteroduplex consisting of the mature miRNA and the passenger strand. The mature miRNA is loaded into an Argonaute protein (ALG-1 or ALG-2), forming the core microRNA-Induced Silencing Complex (miRISC). Through partial base-pairing, the miRNA positions miRISC on a target messenger RNA (mRNA) and the recruitment of AIN-1/2, along with other factors, causes repression of the target *via* deadenylation and/or inhibition of translation.

As the central players of these aging pathways are found, understanding how their expression is regulated emerges as a key problem. This review will focus on a prominent class of post-transcriptional regulators called microRNAs (miRNAs). miRNAs are short (∼22 nucleotides), non-coding RNAs that target messenger RNAs (mRNAs) for degradation or translational repression through sequence-specificity (Figure 1) (Bartel, 2018). To regulate their targets, mature miRNAs need to be loaded into an Argonaute (AGO) protein to form the miRNA-induced silencing complex (miRISC). Once bound to a target mRNA via base-pairing interactions with the miRNA, miRISC recruits RNA degradation factors and translational repressors that down-regulate expression of the targeted mRNA through a variety of mechanisms, some of which are not yet well-understood. In *C. elegans*, there are more than 25 AGOs, but AGO-Like Gene 1 (ALG-1), and ALG-2 are the main AGOs dedicated to the miRNA pathway (Youngman and Claycomb, 2014). These two effector proteins are developmentally redundant and losing both AGOs results in embryonic lethality (Grishok et al., 2001; Vasquez-Rifo et al., 2012). There are well over 100 different miRNA genes in the *C. elegans* genome (Kozomara et al., 2019), and specific miRNAs have been shown to impact important biological processes, ranging from developmental timing (Lee et al., 1993) and neuronal patterning (Johnston and Hobert, 2003) to stress recovery (Pagliuso et al., 2021) and innate immunity (Zhi et al., 2017).

**FIGURE 2:**
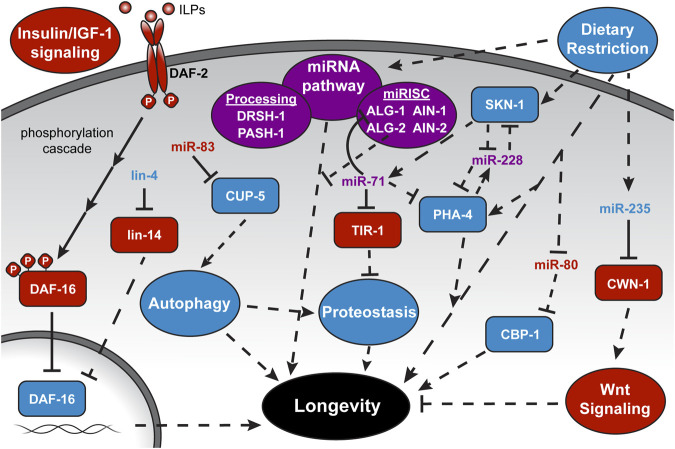
MicroRNA regulation of longevity-associated pathways. Depicted is the complex interplay among miRNAs, targets, and longevity pathways highlighted in this review. More comprehensive schematics and discussion of individual pathways can be found in the recommended reviews in the introduction. ILPs, Insulin-like Peptides, and full gene names are provided at their first mention in the text. Solid lines represent direct interactions. Dashed lines represent indirect or unknown interactions. Pathway names are within ovals, proteins are within rectangles, and miRNAs stand alone. A pathway or factor with a blue background or text color is considered “lifespan-promoting,” while red represents “lifespan-antagonizing,” and purple is ambiguous within the context depicted.

The first reported miRNA with a role in aging in any organism was also the very first discovered miRNA, *C. elegans lin-4* (cell lineage) (Lee et al., 1993; Boehm and Slack, 2005). The *lin-4* gene was originally identified as a key regulator of temporal identity during early larval development (Chalfie et al., 1981; Ambros and Horvitz, 1984). Pioneering work in the Ambros and Ruvkun labs revealed that *lin-4* encodes a tiny non-coding RNA that binds the 3′ UTR of the mRNA encoding the transcription factor *lin-14*, repressing its expression (Lee et al., 1993; Wightman et al., 1993). Down-regulation of *lin-14* by lin-4 miRNA is critical for transition from first to later larval cell fates in several tissues (Olsen and Ambros, 1999). Curiously, the expression of *lin-4* and *lin-14* remains detectable into adulthood, leading Dr. Frank Slack’s group to hypothesize that the pair may have roles beyond development. In support of this idea, they observed that losing the function of *lin-4* significantly reduces lifespan, whereas decreasing *lin-14* expression extends lifespan (Boehm and Slack, 2005). Additionally, they were able to show that this extension is dependent on two transcription factors in the IIS pathway, DAF-16 (dauer formation) and HSF-1 (heat shock factor) (Figure 2). Since then, many more miRNAs have been shown to influence the rate of aging through the regulation of established longevity pathways, and we recommend the following reviews for more in-depth summaries: Kinser and Pincus, 2020; Kim and Lee, 2019; Ambros and Ruvkun, 2018; Uno and Nishida, 2016; Garg and Cohen, 2014; Inukai and Slack, 2013; Smith-Vikos and Slack, 2012; Jung and Suh, 2012. Building upon these works, this review emphasizes recent findings, and methods specifically relating to the miRNA pathway in aging *C. elegans*.

## Opposing miRNA Effects on Aging Through the Insulin/IGF-1 Signaling Pathway

As the first pathway to show significant lifespan extension by genetic manipulation of its core components (Friedman and Johnson, 1988; Kenyon et al., 1993), the IIS pathway has frequently been the center of aging studies. The IIS pathway links nutrient levels to longevity through a phosphorylation cascade activated by insulin-like peptides binding to a transmembrane receptor, DAF-2, which eventually results in the phosphorylation of the Forkhead box O (FOXO) transcription factor DAF-16 (Murphy and Hu, 2013). This phosphorylation restricts DAF-16 from entering the nucleus and accessing its transcriptional targets. Reduced insulin signaling and underphosphorylation of DAF-16 results in its nuclear translocation and transcriptional activation of genes that promote longevity and stress resistance. Consequently, partial loss of function mutations in *daf-2* can double the lifespan of *C. elegans* in a way that is entirely dependent on DAF-16, while losing the function of *daf-16* can substantially reduce lifespan (Kenyon et al., 1993).

Considering the broad requirement for the miRNA pathway in *C. elegans* embryogenesis and larval development (Alberti and Cochella, 2017), a general loss of miRNAs or their ability to function would be expected to negatively impact lifespan. Indeed, depletion of mature miRNA levels by inactivation of the PASH-1 miRNA processing factor or down-regulation of both *alg-1* and *alg-2* together in adult *C. elegans* shortens lifespan (Figure 2) (Lehrbach et al., 2012; Aalto et al., 2018). However, a more complicated role for the miRNA Argonautes was uncovered when they were individually tested for roles in aging. Aalto and colleagues found that loss of *alg-1* shortens while loss of *alg-2* extends lifespan (Aalto et al., 2018). These longevity phenotypes are independent of the roles of *alg-1* and *alg-2* in development, as their depletion by RNAi at the onset of adulthood also results in opposite lifespan effects (Aalto et al., 2018). These aging phenotypes are consistent with the differential mis-regulation of many genes under the transcriptional control of DAF-16. In *alg-2* mutants, over 30% of the up-regulated genes are considered DAF-16 targets and the extended lifespan of these mutants is dependent on *daf-16* activity (Aalto et al., 2018). In contrast, genes regulated by DAF-16 tend to be down-regulated in *alg-1* mutants, and loss of *alg-1* reduces the *daf-2* extended lifespan. Furthermore, loss of both *alg-1* and *daf-16* together shortened lifespan further than either mutant alone, suggesting that *alg-1* influences other modes of longevity regulation in addition to the IIS pathway. This broader role for *alg-1* is consistent with the conclusion that *pash-1* acts in parallel to the IIS, converging on downstream aging-associated genes (Lehrbach et al., 2012). Identification of the specific miRNAs and targets that alter lifespan when mis-regulated is needed to better understand the role of the general miRNA machinery in controlling longevity in *C. elegans.*

One miRNA that has been repeatedly implicated in promoting longevity through the IIS pathway is miR-71. Attention was originally drawn to miR-71 because it was one of the few up-regulated miRNAs in aging *C. elegans* (De Lencastre et al., 2010). A functional role for *miR-71* was established with the demonstration that genetic loss of *mir-71* shortens lifespan and overexpression of miR-71 extends it through pathways dependent on *daf-16* (De Lencastre et al., 2010; Boulias and Horvitz, 2012). Additionally, gene regulatory network analysis places miR-71 as a highly connected node among aging-associated miRNAs, transcription factors, and miRNA biogenesis genes (Inukai et al., 2018). Surprisingly, a direct target of miR-71 in adult *C. elegans* seems to be *alg-1* (Figure 2)*.* The *alg-1* 3′UTR contains two miR-71 binding sites that confer miR-71 dependent repression of *alg-1* in adult animals (Broughton et al., 2016; Inukai et al., 2018). Disruption of this regulatory loop, where miR-71 represses the expression of an Argonaute needed for miRNA function, has broad downstream consequences that might explain how loss of *alg-1* as well as its negative regulator, *miR-71,* both result in similar shortened lifespan phenotypes. Since Argonaute proteins stabilize bound miRNAs, higher levels of ALG-1 in the absence of miR-71 results in global increases in miRNA abundance and, likely as a consequence, changes in protein coding gene expression (Inukai et al., 2018). Studies focused on how variations in miRNA and mRNA abundance in *miR-71* or *alg-1* mutants impact lifespan will further elucidate this intriguing miRNA feedback loop.

## Up-Regulation of miRNA Pathway Factors Promotes Longevity Induced by Dietary Restriction

Altering the caloric intake of an organism is another mode of regulating longevity. Dietary restriction (DR) by limiting food consumption without malnutrition delays aging and age-related diseases in many organisms (Fontana and Partridge, 2015). In *C. elegans*, DR can extend lifespan by up to 70% depending on the regimen, whether it’s chronic, intermittent, or specific nutrient deficient fasting (Honjoh et al., 2009; Kaeberlein et al., 2006; Lee et al., 2006). Different regimens seem to mediate this extension through distinct pathways, requiring downstream effectors from IIS, TOR (target of rapamycin), and AMPK (adenosine monophosphate-activated protein kinase) pathways (Honjoh et al., 2009; Greer et al., 2007). Given the broad role of the miRNA pathway in gene regulation, it is perhaps not surprising that core miRNA factors are required for DR-induced longevity. An intermittent fasting (IF) protocol, where adult *C. elegans* were alternatively fed and starved every 2–3 days, was found to induce widespread transcriptional changes that promote longevity (Uno et al., 2013). Notably, expression of the miRNA processing enzyme DRSH-1 (drosha), as well as miRISC components ALG-1, ALG-2, AIN-1 (alg-1 interacting protein), and AIN-2 were observed to be up-regulated in response to IF (Figure 2) (Kogure et al., 2017). Increased levels of miRNA pathway components may contribute to IF-induced longevity, as the loss of DRSH-1 function entirely inhibits and loss of ALG-1, ALG-2, or AIN-1 partially suppress the lifespan extension. While the levels of several miRNAs also changed in response to IF (Kogure et al., 2017), a direct connection between the miRNA pathway and target genes that underlie the lifespan extension caused by IF is yet to be uncovered.

As another mode of DR, limiting caloric intake by bacterial food dilution extends lifespan by ∼40% and also induces changes in the expression of specific miRNAs (Rollins, 2019; Smith-Vikos et al., 2014; Xu et al., 2019). Down-regulation of two miRNAs, miR-58 and miR-80, may be associated with enhanced translation of specific transcripts, as target sites for miR-58 and miR-80 were enriched in the 3′UTRs of mRNAs that increase in polysome association during DR (Rollins, 2019). Several studies have now shown that expression of miR-80 is regulated by food (Vora et al., 2013; Kogure et al., 2017; Rollins, 2019). Moreover, down-regulation of miR-80 upon food deprivation reflects the DR-constitutive state of *miR-80* loss of function mutants (Vora et al., 2013). The deletion of *mir-80* results in an extended lifespan and healthspan in a pathway that is dependent on the histone acetyltransferase transcriptional coactivator homolog, CBP-1 (CREB-binding protein) (Figure 2) (Vora et al., 2013). While *cbp-1* has potential miR-80 target sites in its 5′UTR and coding sequence, more work is needed to establish whether these non-canonical sites mediate direct repression by miR-80.

DR-induced longevity by reduced caloric intake also depends on specific miRNAs. MiR-71 and miR-228 are up-regulated at the onset of DR in adult *C. elegans,* and loss of these miRNAs prohibits lifespan extension by DR (Smith-Vikos et al., 2014). It has been proposed that miR-71 and miR-228 modulate lifespan together through a feedback loop with transcription factors PHA-4 (pharynx development) and SKN-1 (skinhead), which play integral roles in DR-induced longevity (Bishop and Guarente, 2007; Panowski et al., 2007). How PHA-1 and SKN-1 regulate the expression of these miRNAs and if they themselves are direct targets of these miRNAs are outstanding questions.

Focus on another DR-induced miRNA, miR-235, has revealed a mechanism for promoting longevity by suppressing an antagonistic pleiotropic pathway. Antagonistic pleiotropy refers to properties of a gene that enhance fitness early in life and negatively impact viability at post reproductive stages (Austad and Hoffman, 2018). An example is the Wnt-signaling pathway, which is essential for proper development and metabolism (Clevers, 2006; Sethi and Vidal-Puig, 2010) but also can induce various age-related pathologies when inappropriately active in adults (Ng et al., 2019). Xu et al., found that Wnt-signaling in calorie restricted adults is subdued by the induction of miR-235, and loss of this miRNA abolishes DR-mediated lifespan extension (Xu et al., 2019). A direct target of miR-235 seems to be the Wnt ligand *cwn-1* (*C. elegans* Wnt family)*,* which has a single miR-235 binding site in its 3′UTR. Interestingly, increased expression of miR-235 and suppression of Wnt-signaling was only observed in adults and not in younger stages of animals under DR regimens. Thus, there is temporal control over the ability of miR-235 to respond to DR and modulate Wnt signaling to promote longevity. This apparent switch-like feature prevents premature repression of Wnt signaling early in development. This work provides a foundation for exploring the role of homologs of miR-235 (called miR-92 in mammals) and Wnt-signaling genes in regulating longevity in higher organisms under different nutrient states.

## Cross-Tissue Regulation of Autophagy and Proteostasis by miRNAs

Autophagy is a broadly conserved cellular mechanism for eliminating damaged proteins and organelles and recycling their components (Mizushima, 2007). Autophagy provides a source of nutrients for cellular functions during stress and helps clear misfolded proteins and old organelles from cells (Levine and Kroemer, 2008). Protein homeostasis (proteostasis) depends on autophagy and the ubiquitin-proteosome system (UPS), and these activities decline with age (Sarkis et al., 1988; Taylor and Dillin, 2011; Chang et al., 2017). Accordingly, pathways that impact lifespan, such as IIS, nutrient sensing through TOR, and dietary restriction, and converge onto autophagy to regulate aging (Bareja et al., 2019). Given its central role in multiple longevity pathways, a better understanding of how autophagy genes are regulated may reveal mechanisms for slowing deactivation of this pathway in aging and promoting healthy longevity.

To identify a basis for the decline in autophagy in older *C. elegans,* Zhou et al., 2019, examined transcriptome changes in aging adults. From this, miR-83 stood out as an up-regulated miRNA with target sites in the autophagy-related gene, *cup-5* (coelomocyte uptake-defective) (Zhou et al., 2019). Loss of *miR-83* results in an extended lifespan and enhanced autophagy and proteostasis through derepression of *cup-5* in adults. Surprisingly, age-associated up-regulation of miR-83 seems to only occur in the intestine, yet repression of *cup-5* and the resulting dysregulation of autophagy was observed in intestine as well as in body wall muscle (BWM) cells. The cell non-autonomous regulation of *cup-5* is achieved by transport of miR-83 from the intestine to BWM cells. This remarkable finding establishes a specific role for the miRNA pathway in regulating autophagy during aging, and also presents a compelling example of a secreted miRNA having a biologically relevant function outside of its origin cell. It also questions the purpose of expressing a miRNA that negatively regulates autophagy and, hence, longevity. The answer, again, likely relates to antagonistic pleiotropy (Austad and Hoffman, 2018). Early in life, miR-83 is needed for proper germline development and fertility, and its post-reproductive effects are not subject to selection. The homolog of miR-83, called miR-29 in mammals, has the potential to target autophagy pathway genes (Zhou et al., 2019), raising the possibility that age-related changes in autophagic capacity might also be regulated by the miRNA pathway in humans.

In addition to autophagy, the other major pathway for maintaining proteostasis, UPS, is also under miRNA control. It was recently shown that short lived *miR-71* mutants are defective in ubiquitin-dependent protein turnover (Finger et al., 2019). Reduced lifespan and UPS activity both seem to be due to up-regulation of the miR-71 target *tir-1* (Toll and Interleukin 1 Receptor domain protein)*,* as these phenotypes are largely suppressed by loss of *tir-1* and copied in strains expressing a version of *tir-1* that lacks the miR-71 3′UTR binding sites (Finger et al., 2019)*.* In another example of cross-tissue effects of a miRNA, repression of *tir-1* by miR-71 occurs in AWC olfactory neurons, leading to release of neuropeptides that stimulate UPS activity in the intestine. Additionally, food odor was found to promote proteostasis and longevity through miR-71 mediated regulation of *tir-1* (Finger et al., 2019). The proposal that odor controls the ability of a miRNA to regulate its target offers a new mechanism linking environmental cues to the regulation of programs, such as UPS, that promote longevity.

## New Methods for Elucidating miRNA Expression and Function During Aging

Cell non-autonomous signaling as a modifier of aging has been explored in various model organisms (Miller et al., 2020), but the broad impact of miRNAs on this phenomenon is just beginning to be recognized. Considering the newly identified cross-tissue roles of miR-71 and miR-83 in aging *C. elegans* (Finger et al., 2019; Zhou et al., 2019), it will be critical to determine where a miRNA of interest is expressed, where its targets are being regulated, and which tissues depend on this regulation to control longevity. In the first large scale effort, spatio-temporal control of miRNA expression was studied using reporters consisting of the miRNA promoter fused to GFP (Martinez et al., 2008). Most of the miRNA promoters that were tested showed tissue-specificity, with less than 5% having ubiquitous somatic expression. This tool offers useful information regarding the transcriptional domain of a miRNA gene, with the caveat that regulatory elements might have been excluded in the reporter design. Furthermore, it does not necessarily show the relative levels of mature, active miRNA or where it exerts regulation.

More recently, systematic efforts to isolate and sequence miRNAs from individual tissues have been developed to gain cellular-level resolution of mature miRNA localization. A new technology, microRNome by methylation-dependent sequencing (mime-seq), utilizes expression of a plant-specific methyltransferase, HEN1 (Hua enhancer), in individual tissues to methylate mature miRNAs, which can then be chemoselectively cloned and sequenced (Alberti et al., 2018). This method produces the miRNA profile of whichever cells express HEN1, and it is sensitive enough to reveal the miRNome of just two sensory neurons. Mime-seq is easier and less disruptive than previous tissue-specific isolation methods, like fluorescence-activated cell sorting, laser-capture microdissection, or immunoprecipitation, and has significantly higher yield. Though it detects miRNAs present in a particular tissue, they may not all be active miRNAs, as evidenced by the detection of miRNA passenger strands. To focus on active miRNAs, tissue-restricted miRISC components can be immunoprecipitated. This method was originally developed using tissue specific expression of the miRISC cofactor, AIN-2 (Kudlow et al., 2012). As AIN-2 is generally required for target regulation by miRISC, the isolation of complexes containing this factor are likely to select for functional miRNAs (Zhang et al., 2007). More recently, a spatio-temporal map of active miRNAs was constructed via immunoprecipitation of epitope-tagged ALG-1 or ALG-2 expressed in intestine, body wall muscle, or the nervous system (Brosnan et al., 2021). Preferential association of miRNAs with ALG-1 or ALG-2 globally in adult *C. elegans* had been uncovered (Aalto et al., 2018), but now this relationship can be viewed with a more nuanced, cell-specific focus. Results from these methods can also be compared to expression data from reporters driven by miRNA promoters to potentially reveal miRNAs, like miR-83, that are synthesized in one tissue and secreted to another to regulate specific targets. Using these applications in adults will shape our spatial understanding of aging-associated miRNAs.

With a higher-resolution picture of the miRNome comes the challenge of identifying direct targets of these miRNAs. Methods that use RNA immunoprecipitation (RIP), cross-linking immunoprecipitation with high-throughput sequencing (CLIP-seq), and individual-nucleotide resolution CLIP (iCLIP) of miRISC components (ALG-1, ALG-2, AIN-1, or AIN-2) have been used to detect miRNA targets genome-wide in larval stage *C. elegans* (Zhang et al., 2007; Zisoulis et al., 2010; Grosswendt et al., 2014; Broughton et al., 2016). Occasionally, sequence reads from some of these techniques correspond to a miRNA ligated to its target site (Grosswendt et al., 2014; Broughton et al., 2016). These rare chimeric sequences provide evidence of *in vivo* miRNA-target site interactions that are not confined to computational predictions. Improved methods that enrich for chimeras formed by RNA isolated from tissue-restricted miRISC components in aging *C. elegans* will go far to define specific targets and miRNAs that impact organismal longevity.

As exemplified by several of the studies highlighted here, advances in genome editing provide efficient and precise tools for validating miRNA targets and verifying their relevance to aging (Friedland et al., 2013). CRISPR-Cas9 can be used to knock out individual miRNAs and mutate potential miRNA binding sites in the 3′UTRs of target mRNAs. Using these genetic manipulations together can corroborate evidence that mis-regulation of a specific miRNA target results in a modification of longevity, or any phenotype of interest. Recently, this technology has been used as the basis of a large-scale mutagenesis screen to analyze the regulatory sequences of 3′UTRs (Froehlich et al., 2021). As a proof of concept, hundreds of small deletions were generated along the 3′UTR of *lin-41*, a gene known to be targeted by miRNA *let-7* (lethal) (Reinhart et al., 2000; Vella et al., 2004; Ecsedi et al., 2015). The screen succeeded in identifying the two previously-characterized *let-7* binding sites as important regulatory elements in the *lin-41* 3′UTR. Applying this strategy to aging-related genes may pinpoint specific 3′UTR sequences subject to regulation by miRNAs, as well as aid in untangling the complexity of multiple miRNAs targeting a single gene.

Advances in tissue-specific depletion of a gene of interest provide new means to better understand how a mis-regulated miRNA target contributes to aging phenotypes. Two methods for removal of a specific protein from a particular cell type that utilize GFP-targeted nanobodies or an auxin-inducible degron have been recently developed for *C. elegans* (Zhang et al., 2015; Wang et al., 2017). In the first system, a GFP-targeting nanobody fused to a ubiquitin ligase adaptor is driven by a tissue-specific promoter, which results in degradation of any GFP-tagged proteins expressed in the same tissue (Wang et al., 2017). Thus, spatial but not temporal control is available, as this system drives constitutive depletion of a protein of interest from an individual tissue. Removal of a protein from a specific tissue at a desired time can be achieved with the auxin-inducible degradation (AID) system (Zhang et al., 2015). Tissue-specific expression of the plant Transport Inhibitor Response 1 (TIR1) protein allows for spatially restricted degradation of a protein of interest that is tagged with a degron, which is only recognized by the TIR1-ubiquitin ligase complex in the presence of the plant hormone auxin (Nishimura et al., 2009). This allows researchers to trigger tissue-specific degradation of a degron-tagged protein at any life stage. While caution should be taken to recognize potential unintended effects of the degron tag, TIR1, or the presence of auxin (Schiksnis et al., 2020; Bhoi et al., 2021; Loose and Ghazi, 2021), improvements to the AID system for *C. elegans* promise to make this a powerful tool for studying the role of miRNA targets in aging, while bypassing their potential requirement during development (Ashley et al., 2021; Hills-Muckey et al., 2021; Negishi et al., 2021). A summary of the experimental methods described in this section are outlined in Table 1.

**TABLE 1:** Summary of the methods for elucidating miRNA expression and function that are reviewed in the text.

Areas of miRNA discovery	Experimental methods	Additional information provided	References
Defining miRNA expression	miRNA promoter-GFP fusions	Shows only where miRNA genes are expressed	Martinez et al. (2008)
	mime-seq	Shows mature miRNA localization	Alberti et al. (2018)
		Immunoprecipitation of miRISC factors	Shows active miRNA localization	Kudlow et al. (2012)
				Brosnan et al. (2021)
Identifying miRNA targets	RIP, CLIP seq, iCLIP	Gives mRNAs associated with miRISC components; chimeric reads give direct miRNA-target interactions	Zhang et al. (2007)
			Zisoulis Yeo, (2010)
			Grosswendt et al. (2014)
			Broughton et al. (2016)
	CRISPR-based 3′UTR mutagenesis screen	Tests functionality of miRNA binding sites on target mRNA expression	Froehlich et al. (2021)
Determining miRNA target functions	GFP-targeting nanobodies	Spatial control of target protein degradation	Wang et al. (2017)
	AID system	Spatiotemporal control of target protein degradation	Zhang et al. (2015)
			Ashley et al. (2021)
			Negishi et al. (2021)
				Hills-Muckey et al. (2021)

## Concluding Remarks

As a model organism for uncovering roles for the miRNA pathway in aging, *C. elegans* offer a favorable balance of simple and complex. Besides their ease of maintenance and brief lifespan, established molecular tools and worm-specific resources make hypothesis generation and experimental set-up straightforward. Though *C. elegans* have limited anatomical features compared to mammals, there is now compelling evidence that miRNAs coordinate aging across major tissues in worms. Given the conservation of the miRNA pathway, we expect that specific miRNAs will also have cell non-autonomous functions that modulate longevity in humans. The studies highlighted here contribute not only to a better understanding of the role of the miRNA pathway in aging, but also of mechanisms controlling miRNA expression and targeting, irrespective of the conservation of individual components. Further advances in the field will depend on identifying direct targets of miRNAs that regulate aging, potentially in a spatio-temporal manner. As these regulatory networks are solidified, searching for upstream factors that regulate the aging-associated miRNAs themselves will deepen our understanding of the programming changes that underlie differences in miRNA function in development versus adulthood. With a legacy of leading the discovery of miRNAs and conserved longevity pathways, we anticipate that *C. elegans* will continue to be at the forefront of research into miRNA-mediated regulation of lifespan and inspire new approaches to improve healthspan and treat age-related diseases in humans.
